# Invasive Group A Streptococcal Infections, Clinical Manifestations and Their Predictors, Montreal, 1995–2002

**DOI:** 10.3201/eid1101.030651

**Published:** 2005-01

**Authors:** Maria-Graciela Hollm-Delgado, Robert Allard, Pierre A. Pilon

**Affiliations:** * Unité Maladies Infectieuses, Montréal, Québec, Canada; † McGill University, Montreal, Québec, Canada; ‡ Université de Montréal, Québec, Canada

**Keywords:** Streptococcus pyogenes, streptococcal infections, bacteremia, shock, respiratory tract infections, soft-tissue infections, death, epidemiologic factors, population surveillance, Quebec, research

## Abstract

We identified 306 invasive group A streptococcal infections (IGASI) by passive population-based surveillance in Montreal, Canada, from 1995 to 2001. The average yearly reported incidence was 2.4 per 100,000 persons, with a 14% death rate. Among clinical manifestations, incidence of pneumonia increased from 0.06 per 100,000 in 1995 to 0.50 per 100,000 in 2000. Over a span of 7 years, the odds of developing pneumonia increased (odds ratio [OR] = 1.21, 95% confidence interval [CI] 1.0–1.5), while they decreased for soft-tissue infections (OR = 0.86, 95% CI 0.7–1.0). Serotypes M1 and M3 accounted for 30% of IGASI. However, neither serotype was significantly associated with specific clinical manifestations, which suggests that manifestation development among IGASI might be attributable to host or environmental factors rather than the pathogen. In our study, these factors included age, gender, underlying medical conditions, and living environment, yet none explained temporal changes in risk for pneumonia and soft-tissue infections.

## Abstract 2

Specific clinical manifestations of invasive group A streptococcal infection appear to develop not in response to the pathogen, but rather to host or environmental factors.

Since the mid-1980s, concern has grown that invasive group A streptococcal infections (IGASI) have been increasing in incidence and severity (*1**–**3*). In particular, the emergence of streptococcal toxic shock syndrome (STSS) during the 1980s is frequently cited as an example of increasing severity (*4*).

Person-to-person transmission of *Streptococcus pyogenes* (the causative agent for IGASI) primarily occurs through respiratory droplets, although it may also spread through body secretions from an infected patient (*5**,**6*). Additionally, M serotypes of *S. pyogenes* that cause severe disease in a patient are more likely to cause severe disease in subsequent patients (*6*). These serotypes include 3 (M1, M3, and M18) that are strongly associated with pathogenicity (*7*). Nonetheless, some evidence indicates that persons in whom IGASI from the same strain of *S. pyogenes* develops may have different clinical manifestations of this disease (*8**,**9*). Other risk factors for IGASI include patient’s age and underlying medical conditions (e.g., varicella). However, what factors may be associated with different clinical manifestations of IGASI is unclear (*10**–**22*).

Some studies have examined the role of age, varicella, and chronic conditions such as diabetes mellitus and alcoholism as predictors for necrotizing fasciitis, soft-tissue infections, and STSS (*21**–**24*), yet little is known regarding other IGASI determinants. In this study, we describe the status of both IGASI and their clinical manifestations on the island of Montreal. We also identify predictors for clinical manifestations and death due to IGASI, which could explain temporal fluctuations in the incidence and severity of this disease.

## Methods

### Surveillance of IGASI

Data used in our study were collected during passive surveillance of IGASI among all residents of the island of Montreal (population = 1.8 million: 21,529 births per year from 1996 to 1999 [*25*]). Cases that occurred and were reported from January 1, 1995 (the year IGASI became a notifiable disease in the province of Quebec), through February 28, 2002, were included in our study.

Once the public health department had been notified of a potential case, usually by a hospital laboratory, a 6-part questionnaire was completed by using information from the physician or infection control nurse of the health center where the case-patient was identified or treated. Questions included the patient’s demographic information, general medical information, laboratory results, diagnostic criteria, and medical history before the IGASI. With this information, all IGASI were classified into 1 of 3 groups: confirmed cases (*S. pyogenes* isolated from a normally sterile site), clinical cases (*S. pyogenes* isolated from a nonsterile site and toxic shock not attributable to any other cause), or noncases. Data on confirmed and clinical cases were entered into the regional notifiable infections computer database. This database was used for our study.

### Laboratory Assessment of IGASI Isolates

Initial laboratory confirmation of *S. pyogenes* was made by using standard methods (*26*). Isolates were then collected and sent to the Canadian National Center for Streptococcus in Edmonton for further testing of the opacity factor, as well as M, T, and R surface proteins. The methods have been described in detail elsewhere (*27*). Briefly, antiopacity factor (AOF) typing was performed on any positive opacity factor sample. Although AOF testing does not possess the same type specificity as M typing, it is frequently used because of difficulties in producing antisera for certain M serotypes. Its use has been validated for most strains identified in industrialized countries. However, since 2000, the national center has supplemented AOF testing with *emm* gene sequencing for some nontypeable M serotype samples. Data on these results were not available for this study.

### Classification of Outcomes

For our study, we looked at 5 dichotomous outcomes: STSS, soft-tissue infections, bacteremia, pneumonia, and death attributable to IGASI. All were invasive and defined in accordance with the classification of group A streptococcal infections (*28*).

STSS was defined according to the 1993 Working Group on Severe Streptococcal Infections consensus definition for a probable or confirmed case (*28*). Soft-tissue outcomes included fasciitis, myositis, cellulitis, or erysipelas. Bacteremia was characterized by a positive hemoculture, without any source of infection. Pneumonia attributable to IGASI was based on a clinical diagnosis made by the treating physician and could include STSS with respiratory distress.

### Classification of Independent Variables

Age, calendar month, and year in which the IGASI case occurred were included in our study as continuous variables. Gender (male or female); underlying medical conditions (drug use, alcohol abuse, varicella, prior trauma or wound, cancer, and immunosuppression); type of living environment (hospital, daycare or preschool, school, work, other, and not available); as well as M, T, and R surface protein serotypes (presence or absence of a specific serotype) were all included as dichotomous variables. For those serotypes with identical strength of association with a given outcome, a single new dichotomous variable was created to represent the presence of one or the other (e.g., presence of either serotype M12 or M28 versus absence of both serotypes). Finally, since predominant site of infection (bacteremia, fasciitis, cellulitis or erysipelas, myositis, peritonitis, respiratory manifestations, septic arthritis, and other) was partially used in distinguishing between bacteremia, pneumonia, and soft-tissue infections, this variable was only considered a covariate of interest in models with STSS and death as their outcomes.

### Statistical Analysis

The incidence (per 100,000), death rate attributable to IGASI, and proportion of IGASI cases due to a specific clinical manifestation were estimated by using data collected from 1995 through 2001. Incidence and proportion estimates were not calculated for 2002, given that only 2 months of data were available. Projected annual population estimates for Montreal were used when calculating the reported annual incidence of IGASI (*25*). Incidence and proportion of IGASI cases stratified by gender, calendar year, and age group were then calculated. Finally, temporal trends were assessed by using the chi-square test for trend.

For the inferential component of our study, we conducted unconditional logistic regression with SAS version 8.0 (SAS Institute, Cary, NC, USA). This test was initially performed by including in the model variables with a univariate likelihood ratio p value < 0.20. Among these factors, those with the highest multivariate Wald chi-square p value were then individually dropped, until the lowest Akaike Information Criterion value was attained. The McGill University Faculty of Medicine Institutional Review Board approved the study.

## Results

**Figure 1:**
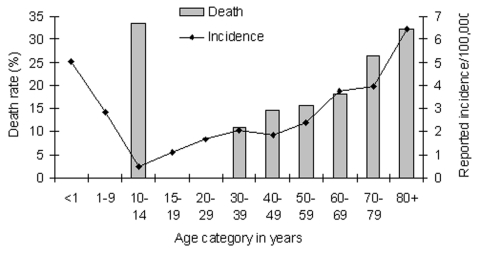
Annual incidence and death rate of invasive group A streptococcal infections, by age, in Montreal, Canada, 1995–2001.

From 1995 through 2001, a total of 306 cases of IGASI were reported on the island of Montreal. The incidence of IGASI rose from 1.05 per 100,000 (19 cases) in 1995 to 1.71 (31 cases) in 1996 and 3.32 (60 cases) in 1997. After 1997, the incidence appeared to stabilize: 2.77 (50 cases) in 1998, 2.50 (45 cases) in 1999, 3.21 (58 cases) in 2000, and 2.37 (43 cases) in 2001. The average annual incidence of IGASI was 2.4 per 100,000. Most IGASI cases occurred in persons >40 years of age (172 [56%] of 306 cases) (Figure 1). The median age of patients was 46 years (range 1.5 months to 92 years).

Of the 306 reported IGASI cases, 112 (37%) were soft-tissue infections, 84 (28%) bacteremia, 32 (10%) pneumonia, and 29 (10%) STSS. Among patients with soft-tissue infections, 6 (5%) of 112 cases had myositis, 31 (28%) had cellulitis, and 76 (68%) had necrotizing fasciitis; 1 patient had both cellulitis and necrotizing fasciitis. We did not identify any significant trend over time with regard to the proportion of different clinical manifestations. As for specific clinical manifestations of IGASI, we estimated that bacteremia occurred, on average, in 0.66 per 100,000 persons each year, STSS in 0.23 per 100,000, soft-tissue infections in 0.89 per 100,000, and pneumonia in 0.25 per 100,000.

The predominant M serotypes included M1 (22%), M3 (12%), M28 (9%), M12 (8%), M4 (6%), and M6 (4%). Remaining serotypes accounted for <3% of isolates. Twenty percent of samples were nontypeable.

### Pneumonia

**Figure 2:**
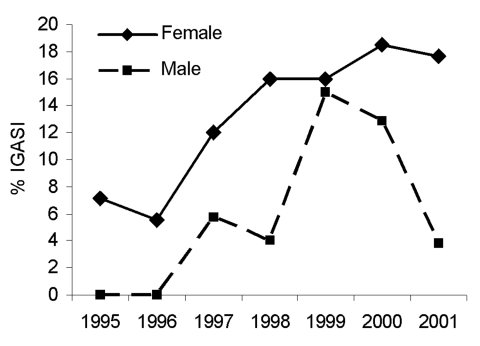
Pneumonia as a proportion of invasive group A streptococcal infections (IGASI) by gender, Montreal, Canada, 1995–2001.

The incidence of pneumonia appeared to significantly increase over time (χ^2^ = 5.65, p = 0.018), with an annual incidence of 0.06 per 100,000 in 1995 and 1996, 0.28 in 1997 and 1998, 0.39 in 1999, 0.50 in 2000, and 0.22 in 2001. This finding was confirmed by the odds of having pneumonia significantly increasing with each successive calendar year (adjusted odds ratio [aOR] = 1.21, 95% confidence interval [CI] 1.0–1.5). The proportion of women and girls with pneumonia (Figure 2) also significantly increased (χ^2^ = 5.03, p = 0.025), with women more likely to have pneumonia as compared to men (aOR 2.20, 95% CI 1.0–4.9). Gender was not associated with year in which the case occurred.

### STSS

We did not detect a significant secular trend in the occurrence of STSS (χ^2^ = 0.54, p = 0.46). Persons who abused alcohol (aOR 7.66, 95% CI 1.9–30.3]), were infected with serotype M9 (aOR 39.98, 95% CI 1.9–836]), or who had fasciitis (aOR 10.21, 95% CI 4.1–25.7]) were at a significantly greater risk of having STSS.

### Soft-tissue Infections

No significant secular trend was apparent in the incidence of soft-tissue infections (χ^2^ = 0.48, p = 0.49). However, the odds of developing this manifestation as opposed to another significantly decreased with each successive calendar year (aOR 0.86, 95% CI 0.7–1.0). Drug use was weakly associated with soft-tissue infections (unadjusted OR 1.86, 95% CI 0.8–4.4). Given that trauma was a significant univariate risk marker for soft-tissue infections (OR 2.78, 95% CI 1.6–4.8), this association might have been attributable to injection drug use resulting in a trauma or wound. However, in our study, no correlation was seen between drug use and trauma (*r* = 0.002, p = 0.97). Furthermore, the association between drug use and soft-tissue infections became significant after adjusting for trauma or wound (OR 2.83, 95% CI 1.0–8.0).

Varicella and serotypes M6, M12, or M22 were significant predictors for developing soft-tissue infections with aORs of 5.69 (95% CI 1.4–23.1), 4.3 (95% CI 1.1–16.7), 9.1 (95% CI 1.3–64.5), and 27.9 (95% CI 2.7–289), respectively. None of these factors were correlated with calendar year.

### Bacteremia

The incidence of bacteremia did not appear to change over time (χ^2^ = 0.56, p = 0.45). Only protective factors against bacteremia were identified: attending a school (aOR 0.15, 95% CI 0.0–0.7) and trauma or wound (aOR 0.4, 95% CI 0.2–0.9).

### Death Due to IGASI

The death ratio from IGASI was 15% (42 deaths among 306 cases). The highest proportion of known deaths was among patients with pneumonia (38%, 12 deaths among 32 pneumonia cases), followed by STSS (35%, 10 among 29), bacteremia (17%, 14 among 84), and soft-tissue infections (10%, 11 among 112). Within soft-tissue infections, necrotizing fasciitis had the highest risk for death among all age groups (16%, 5 deaths among 31 cases) followed by cellulitis and erysipelas (8%, 6 among 76). For myositis, among the 6 cases identified during a 7-year period, no deaths were recorded. No secular trends for death ratios were seen for any of the clinical manifestations of IGASI. Among those who died of IGASI, the most common serotypes were M1 (34%) and T1 (30%); however, neither was significantly associated with death (unadjusted OR for M1: 1.86, 95% CI 0.9–4.0; T1: 1.91, 95% CI 0.9–4.1). Predictors for death, after adjustment, are presented in the Table.

**Table:** Adjusted odds ratio (OR) for factors associated with death attributable to invasive group A streptococcal infections, Montreal, Canada, 1995–2002

Variable	OR (95% CI)*
Age (y)	1.04 (1.0–1.1)†
Underlying medical conditions	
No cancer	Referent
Cancer	4.14 (1.6–10.5)
Primary site of infection	
Not cellulitis	Referent
Cellulitis	0.38 (0.1–1.0)
Not pneumonia	Referent
Pneumonia	3.62 (1.4–9.0)
Living environment	
Not working or living in hospital	Referent
Working or living in hospital	3.71 (1.0–13.6)
M serotypes	
Not M2	Referent
M2	10.69 (0.5–220)

*CI, confidence interval. †Increase in risk per increase in year of age.

## Discussion

When the results of our study are examined, several methodologic considerations must be taken into account. First, given that the administration of questionnaires for this study was not standardized, nondifferential misclassification could explain why certain factors in this study were not identified as potential markers for clinical manifestation outcomes.

An additional limitation of our study was the low statistical power. For some measures of association, the probability of detecting a true association was estimated to be as low as 3%. As a result, while this study can identify potential predictors, it cannot exclude them.

Additionally, given that this study was to a certain extent hypothesis-generating, some of the predictors found in this study (particularly those with weak associations) may have occurred by chance. Considering that an α level of 0.05 was used when testing ≈200 associations, at least 10 significant factors would be expected to be identified by chance. In our study, we identified 25 factors to be significantly associated with specific IGASI manifestations.

IGASI and STSS may be increasing in both incidence and severity (*4*). In particular, increasing trends in the IGASI incidence in the United States have been recorded in several hospital-based studies (*29*). Furthermore, past European studies noted a general increase in the incidence, although little evidence shows a trend occurring in the United States (*14**,**30**–**32*). While we documented a trend in the annual incidence of IGASI in Montreal during the first 3 years of our study, the incidence stabilized from 1997 onwards, which suggests that an initial rise in incidence might be attributable to underreporting immediately after IGASI became a notifiable disease. During the 7 years of our study, mortality did not appear to significantly change. Additionally, we could not identify any significant trends in the incidence and mortality of STSS. We did, however, ascertain that pneumonia attributable to IGASI significantly increased during 6 of the 7 years of our study. This finding was particularly evident among women. Our findings agree with those of a study in Ontario, which identified an increasing trend for pneumonia attributable to GAS from 1992 to 1999 (*33*).

To the best of our knowledge, no research has been published on transmission rates for the different clinical manifestations of IGASI. However, the primary mode of person-to-person transmission of *S. pyogenes* is through respiratory droplets (*5**,**6*). Additionally, *S. pyogenes* that causes severe disease in one patient is more likely to cause severe disease in subsequent patients (*6*). Considering these previous study findings, one could hypothesize that secondary contacts of patients with respiratory manifestations might be more likely to acquire an infection leading to severe disease, compared to contacts of patients with other IGASI manifestations.

Even though IGASI is a reportable disease, our results for pneumonia may be an underestimate of the true values. Given that <1% of community-acquired pneumonia is attributed to *S. pyogenes* (*34*), pneumonia caused by this bacterium may have been ascribed to other causes and hence not reported. Our findings are further complicated by difficulties in defining pneumonia (*33*). No standard clinical definition distinguishes IGAS pneumonia from respiratory distress caused by STSS. Although both clinical manifestations might differ with regard to pathophysiology, given that prophylaxis is required for secondary contacts of either manifestation in Quebec, difficulties in distinguishing between these manifestations will probably not affect the public health implications of our findings.

With regard to the generalizability of our results, when comparing our findings with previously published studies, we did not detect any geographic differences in the incidence of IGASI (*17*). Our data showed that the yearly incidence of IGASI in Montreal (1.0–3.3 per 100,000) was similar to the incidence of IGASI in British Columbia (*20*), Ontario (*16*), Israel (*35*), Sweden (*19**,**22*), and the United States (*14**,**18*). Furthermore, death rates from IGASI in Montreal were comparable to death rates calculated for British Colombia (*20*) and Sweden (*19**,**22*). Only Arizona appeared to have a higher death rate due to IGASI, at 20% (*3*). This difference might be attributable to the elevated prevalence of diabetes (a risk factor for IGASI) in the Arizona community studied (*3*).

Along with these descriptive findings, we identified several factors associated with clinical manifestations of IGAS and associated death. Having varicella before IGASI increased the risk of developing a soft-tissue infection 6 times and the risk of dying 5 times. Although we could not identify any literature linking varicella infection with soft-tissue infections, given that soft-tissue infections are the predominant clinical manifestation of IGASI, our findings support previous research that suggests that varicella might be an important risk factor for developing IGASI (*16**,**36*).

Soft-tissue infections were almost twice as likely to develop in persons using drugs. This association could be attributable to injection drug use; however, it remains even after controlling for trauma. One explanation for this unexpected finding could be nondifferential misclassification. A subanalysis of drug use showed that 23% of patients indicated a trauma or wound. However, we were unable to determine the reliability of reporting. While a patient could have affirmatively answered to drug use, a wound inflicted by intravenous drug use may not have been considered sufficiently severe to indicate a trauma or wound.

Our descriptive analysis appears to support previous research findings that those <1 year of age and those >60 years of age have the highest incidence of IGASI (*3**,**21**–**23*). However, previous studies also suggest that children might have a lower incidence of STSS and be at a decreased risk of dying of IGASI (*21*). This research includes a study that identified a nonsignificant 5-fold rise in risk for death per year increase in age. In contrast, our study showed a 2%–4% increase.

Our study finding that M1 and M3 accounted for >30% of all isolates tested for M surface proteins was consistent with previous studies that reported these 2 M serotypes as the most common for IGASI (*3**,**15**,**16**,**23**,**24*). This finding is also consistent with the choice of serotypes to include in streptococcal vaccines being evaluated at the moment. The hexavalent vaccine (*37*) is composed of serotypes *1**, **3**, **5**, **6**, **19**,* and *24*; these types represent 38% of isolates in our study. The types in the 26-valent vaccine (*38*) represent 70% of our isolates. This number includes M1 with 22%; M3 with 12%; M28 with 9%; M12 with 8%; M6 with 4%; M22 and M11 each with 3%; M89 with 2%; and M75, M2, M77, M43, M5, M76, and M33 each with 1%.

Furthermore, our study confirmed univariate model findings from a study by O’Brien et al. that found M3 to be significantly associated with STSS (*14*). However, this association did not remain in our multivariate analysis. Our findings would thus appear to concur with those of a another (case-control) study that found while M1 and M3 may be significant risk factors for IGASI, once a person is infected, environmental and host factors might have a role in determining the type of invasive disease manifestations (*8**,**9*). This finding could explain why IGASI may develop in patients infected with the same strain of GAS but have different clinical manifestations of the disease (e.g., STSS versus pneumonia) (*9*). Future epidemiologic studies of risk factors for clinical manifestations of IGASI might be designed to look at risk factors separately in patients identified with M1 and M3 serotypes. By doing so, nondifferential misclassification might be minimized and risk factors with weaker associations might be more easily identified.
